# Cognitive Impairment, Depression, Comorbidity of the Two and Associated Factors among the Early Sixties in a Rural Korean Community

**DOI:** 10.1371/journal.pone.0079460

**Published:** 2013-11-13

**Authors:** Boyoung Park, Jonghan Park, Jae Kwan Jun

**Affiliations:** 1 National Cancer Control Institute, National Cancer Center, Goyang, Korea; 2 Department of Psychiatry, Catholic University of Daegu, Daegu, Korea; Cardiff University, United Kingdom

## Abstract

This study was conducted to investigate the prevalence of cognitive impairment, depression, and comorbidity of the two conditions and related factors in subjects aged in early 60s. This cross-sectional study included 3,174 inhabitants aged 60–64 years old in a rural area of Korea. Cognitive function was evaluated by the Korean version of the Mini-Mental State Examination (MMSE-K), and depression was measured using the short form of the Geriatric Depression Scale (GDS-15). The overall prevalence of cognitive impairment (MMSE-K≤24) was 17.4%, that of depression was 26.0% (GDS-15≥8), and the co-morbidity was 7.1%. Female gender, living with one housemate, and high GDS-15 score were significantly associated with increased cognitive impairment. Employment status and more years of schooling were associated with a decreased probability of cognitive impairment. Increased depression was significantly associated with bereavement and receiving benefits from the Medical Aid Program. Employed status, more years of schooling, and higher MMSE-K scores were significantly associated with decreased depression. The risk of comorbidity was associated with bereavement and receipt of Medical Aid benefits (odds ratio[OR], 1.85; 95% confidence interval[CI], 1.26–2.71; OR, 5.02; 95% CI, 2.37–10.63; respectively). Employment and more years of schooling were associated with a lower risk of comorbidity (OR, 0.46; 95% CI, 0.34–0.62, *P*-trend <0.01). The correlated factors for cognitive impairment, depression, and comorbidity of the two conditions were similar, and employment status and years of schooling were associated with all three conditions.

## Introduction

The proportion of the world’s population in older ages continues to rise because mortality is delayed in the elderly and fertility remains low. Thus, geriatric mental health is emerging as a major health problem. Cognitive impairment and depression are major mental health problems affecting older people [1], [2]. In Korea, about 10% of elderly people suffer from dementia, and the reported prevalence of major depressive disorder ranges from 4.2% to 9.1%, although there are differences among studies [3]. Factors associated with cognitive impairment and depression have been separately studied by many investigators for decades. However, studies of the factors associated with comorbidity of cognitive impairment and depression are rare, although considerable overlap is evident between the two conditions [3]-[5]. Each condition exacerbates the other. Especially, cognitive impairment worsens depression [6], [7]. When it is considered that individuals with both depression and cognitive impairment are at increased risk for adverse medical, psychological, and cognitive outcomes [8], it is clear that risk factors for comorbidity should be defined, to aid in the maintenance of health in the elderly population.

Although most developed countries have accepted that attainment of a chronological age of 65 years renders a person “elderly” or older, the United Nations uses a cutoff of 60 years. However, most studies of the prevalence of cognitive impairment, depression, and their associated factors have targeted people aged ≥65 years. Aging is a gradual progression and there is no distinct demarcation at age 65 years but studies on cognitive impairment and depression focusing on subjects aged in their early 60s are rare. Only a few studies have targeted the mental health of subjects aged in their early 60s (60–64 years) [9]–[12]. Furthermore, these studies focused on the transition of mental health after age 65[9], [10] or mild cognitive impairment as a prodromal phase of dementia [11], [12]. When it is considered that persons in their early 60s experience many social, physical, and emotional changes, triggered by retirement and other lifestyle changes, mental health and associated factors should be investigated in such persons.

Therefore, in this study, we investigated the prevalence of cognitive impairment and depression and related factors in subjects aged in their early 60s living in a rural area of Korea. We also investigated the factors associated with the comorbidity of cognitive impairment and depression through an exploratory study.

## Materials and Methods

### Study population

Participants in this study were recruited from all inhabitants aged 60–64 years living in Dalseong County, a rural area located in Deagu, South Korea. This area covers 426.92 km^2^ and had 144,487 residents at the end of 1999. Of the 5,015 people aged 60–64 years identified through the national resident registration list, 1,222 were excluded. Among those excluded, 784 had moved outside the city before the study started, 34 were institutionalized, 13 had unknown addresses, and 391 were ineligible due to inaccurate birth-date records. Face-to-face interviews were conducted by trained medical doctors and students. The subjects were given full information about the survey, and those who agreed to participate were interviewed. Among the 3,793 eligible in the population, 402 refused to participate, 191 were absent for all of the three visits, and 26 could not participate due to medical conditions. Of the latter, 21 were hospitalized with acute diseases, 3 were deaf, and 2 had aphasia. Thus, 3,174 people participated in the study, for an overall response rate of 83.7%. We additionally excluded 133 participants who did not complete both tests for cognitive function and depressive symptoms, so data of 3,041 participants were finally analyzed.

### Instruments

Cognitive function was evaluated by the Korean version of the Mini-Mental State Examination (MMSE-K). The instrument was developed to assess cognitive function in older Koreans and scores were adjusted to consider educational level as suggested by Park et al [13]. Participants with a score ≤24 were defined as cognitively impaired [13], [14]. Depression was measured using the short form of the Geriatric Depression Scale (GDS-15). A score ≥8 was suggested as probable clinical depression in the Korean population, although a cutoff score of 6 has been applied in other populations [15], [16]. These two tools were well validated in the Korean population in previous studies. Sociodemographic factors such as gender, marriage, years of schooling, current employment status, type of medical insurance, and number of housemates were recorded. Data on smoking and drinking were also collected.

### Ethics statement

This study was approved by the institutional review board of the Catholic University of Deagu Medical Center. All participants provide verbal informed consent because nearly 35% were never educated and 22% were illiterate, therefore to obtain written consent form from all participants was impossible. Also we considered that it took nearly half hour to finish measurements and those who completed agreed to participate with their own will. The institutional review board of the Catholic University of Deagu Medical Center approved this consent procedure.

### Statistical analysis

Characteristics of the study participants are presented as numbers and proportions, and means and standard deviations of the corresponding MMSE-K and the GDS-15 scores were calculated. Also, we calculated Pearson’s correlation coefficient between MMSE-K and GDS-15 scores. Factors associated with cognitive impairment and depression were initially investigated using univariate analysis. Multivariate logistic regression followed, after adjustment of all variables included in univariate analysis. The independent variables included in analysis were gender, marital status, number of housemates, current employment status, years of schooling, current smoking status, current drinking status, type of medical insurance held, and MMSE-K score (measuring depression) or GDS-15 score (assessing cognitive impairment). We next used multivariate multinomial logistic regression to identify factors associated with four levels of morbidity: (a) no cognitive impairment and no depression; (b) depression only; (c) cognitive impairment only; and, (d) both cognitive impairment and depression. We adjusted for all covariates. All statistical analyses were performed using SAS software (version 9.1; SAS Institute Inc, Cary, NC)

## Results

The basic characteristics of the 3,041 participants who completed both the MMSE-K and GDS-15 and the mean scores on these two scales are presented in Table 1. Approximately 57% of the participants were female (*n*  =  1,743), and 34.4% had never been schooled (*n*  =  1,047). Approximately 17% exhibited cognitive impairment (MMSE-K ≤24), and 26.0% evidenced depression (GDS-15 ≥8). Overall, 63.7% (*n*  =  1,937) showed neither cognitive impairment nor depression, 18.9% (*n*  =  574) showed only depression, 10.3% (*n*  =  314) presented only cognitive impairment, and 7.1% (*n*  =  216) exhibited both cognitive impairment and depression. MMSE-K and GDS-15 scores were correlated, with a correlation coefficient of –0.25 and a P-value <0.01, suggesting a positive association between better cognitive function and fewer depressive symptoms.

**Table 1:** Basic characteristics study participants and scores of the Korean version of Mini-Mental State Examination (MMSE-K) and Geriatric Depression Scale.

Characteristic	Total participants (N = 3041)	MMSE-K	Geriatric Depression Scale
	N (%)	Mean (SD)	Mean (SD)
Gender			
Male	1298(42.7)	27.7(2.9)	4.1(3.9)
Female	1743(57.3)	26.5(3.3)	5.5(4.3)
Marital status			
Married	2361(77.6)	27.2(3.0)	4.5(4.0)
Single/widowed/divorced	680(22.4)	26.3(3.7)	6.2(4.5)
Number of housemates (missing in 1)			
0	242(8.0)	26.6(3.3)	6.3(4.2)
1	1279(42.1)	27.0(3.2)	4.7(4.1)
2	627(20.6)	27.4(3.0)	4.7(4.1)
3 or more	892(29.3)	26.9(3.3)	4.8(4.2)
Employment status (missing in 1)			
Unemployed	988(32.5)	26.7(3.6)	5.7(4.5)
Employed	2052(67.5)	27.2(3.0)	4.5(4.0)
Schooling			
No	1047(34.4)	26.0(3.4)	6.4(4.3)
1–6 years	1362(44.8)	27.1(3.2)	4.4(4.0)
7–9 years	294(9.7)	28.3(2.4)	3.4(3.5)
10 years or more	338(11.1)	28.7(1.5)	2.9(3.2)
Smoking			
Currently non-smoker	2093(68.8)	26.9(3.2)	4.9(4.2)
Current smoker	948(31.2)	27.3(3.2)	4.8(4.2)
Drinking (missing in 1)			
Currently non-drinker	2011(66.1)	26.9(3.2)	5.0(4.2)
Current drinker	1029(33.8)	27.3(3.2)	4.5(4.1)
Medical insurance (missing in 12)			
National Health Iinsurance	2948(96.9)	27.1(3.1)	4.7(4.1)
Medical Aid Program	81(2.7)	25.8(4.5)	9.1(4.8)

Table 2 shows the characteristics associated with the presence of cognitive impairment in the univariate and multivariate analysis. Female gender, living with one housemate, and high GDS-15 score were significantly associated with cognitive impairment, otherwise being employed and having been schooled were associated with a decreased probability of cognitive impairment after adjusted all the variables included in the univariate analysis.

**Table 2:** Associations between socio-demographic variables and cognitive impairment in adults aged 60-64 living in a rural area, Korea.

Characteristic	Univariate analysis	Multivariate analysis
	Odds ratio (95% CI)	Odds ratio (95% CI)
Gender		
Male	1	1
Female	2.71(2.19–3.35)*	1.72(1.26–2.34)*
Marital status		
Married	1	1
Single/widowed/divorced	1.77(1.44–2.18)*	1.17(0.89–1.52)
Number of housemates		
0	1	1
1	0.85(0.60–1.19)	1.52(1.01–2.30)*
2	0.71(0.49–1.04)	1.41(0.90–2.19)
3 or more	0.85(0.60–1.22)	1.37(0.92–2.04)
*P-trend*	0.46	0.58
Employment status		
Unemployed	1	1
Employed	0.67(0.55–0.81)*	0.77(0.63–0.95)*
Schooling		
No	1	1
1–6 years	0.49(0.40–0.60)*	0.64(0.52–0.80)*
7–9 years	0.15(0.09–0.25)*	0.24(0.14–0.42)*
10 years or more	0.08(0.04–0.15)*	0.14(0.07–0.27)*
*P-trend*	<0.01	<0.01
Smoking		
Currently non-smoker	1	1
Current smoker	0.71(0.58–0.88)*	1.02(0.79–1.33)
Drinking		
Currently non-drinker	1	1
Current drinker	0.74(0.60–0.91)*	1.19(0.92–1.53)
Medical insurance		
National Health Insurance	1	1
Medical Aid Program	1.68(1.01–2.79)*	1.10(0.64–1.90)
GDS-15		
1 score increase	1.12(1.10–1.14)*	1.08(1.06–1.11)*

* P-value<0.05.

Factors significantly associated with depression were having no spouse and using the Medical Aid Program. Employment, more years of schooling, and higher MMSE-K score were associated with a decreased rate of depression after adjusted all the variables included in the univariate analysis (Table 3).

**Table 3:** Associations between socio-demographic variables and depression in adults aged 60–64 living in a rural area, Korea.

Characteristic	Univariate analysis	Multivariate analysis
	Odds ratio (95% CI)	Odds ratio (95% CI)
Gender		
Male	1	1
Female	1.96(1.65–2.32)*	0.99(0.76–1.29)
Marital status		
Married	1	1
Single/widowed/divorced	2.25(1.88–2.70)*	1.45(1.13–1.85)*
Number of housemates		
0	1	1
1	0.48(0.36–0.64)*	0.89(0.62–1.28)
2	0.50(0.36–0.68)*	0.96(0.65–1.42)
3 or more	0.53(0.40–0.72)*	0.84(0.59–1.19)
*P-trend*		0.46
Employment status		
Unemployed	1	1
Employed	0.57(0.48–0.67)*	0.63(0.53–0.76)*
Schooling		
No	1	1
1–6 years	0.43(0.36–0.51)*	0.51(0.42–0.62)*
7–9 years	0.26(0.19–0.37)*	0.35(0.24–0.52)*
10 years or more	0.21(0.15–0.29)*	0.30(0.20–0.44)*
*P-trend*	<0.01	<0.01
Smoking		
Currently non-smoker	1	1
Current smoker	0.88(0.73–1.05)	1.12(0.89–1.40)
Drinking		
Currently non-drinker	1	1
Current drinker	0.78(0.65–0.93)*	0.94(0.75–1.17)
Medical insurance		
National Health Insurance	1	1
Medical Aid Program	6.03(3.77–9.65)*	4.43(2.66–7.38)*
MMSE-K		
1 score increase	0.87(0.85–0.89)*	0.90(0.88–0.93)*

* P-value<0.05.

Multivariate multinomial logistic regression analyses showed significant associations of marital status, employment state, years of schooling, type of the medical insurance with comorbidity of cognitive impairment and depression (Table 4). The odds ratio (OR) of single/widowed/divorced participants was 1.85 (95% CI 1.26–2.71) relative to married participants, and that of being employed was 0.46 (95% CI 0.34–0.62) compared with those who were not employed. More schooling was associated with less comorbidity (*P*-trend < 0.01), and receipt of Medical Aid Program benefits was associated with a greater likelihood of comorbidity (OR 5.02, 95% CI 2.37–10.63). Females tended to experience cognitive impairment (only) more than males. Marital and employment status, and type of medical insurance held, were factors associated with the presence of depression (only). Year of schooling was the factor associated with the presence of only cognitive impairment or only depression.

**Table 4:** Multivariate multinomial logistic regression analysis between socio-demographic variables and comorbidity of cognitive impairment and depression in adults aged 60–64 living in a rural area, Korea.

Characteristic	Comorbidity of cognitive impairment and depression	Only cognitive impairment	Only depression
	(n = 216)	(n = 314)	(n = 574)
	Odds ratio (95% CI)	Odds ratio (95% CI)	Odds ratio (95% CI)
Gender			
Male	1	1	1
Female	1.48(0.92–2.38)	1.90(1.29–2.81)*	1.03(0.77–1.38)
Marital status			
Married	1	1	1
Single/widowed/divorced	1.85(1.26–2.71)*	1.11(0.78–1.57)	1.41(1.06–1.87)*
Number of housemates			
0	1	1	1
1	1.36(0.77–2.41)	1.54(0.88–2.68)	0.92(0.61–1.38)
2	1.68(0.91–3.07)	1.10(0.60–2.03)	0.85(0.55–1.32)
3 or more	1.03(0.59–1.81)	1.50(0.87–2.58)	0.91(0.61–1.35)
*P-trend*	0.63	0.82	0.64
Employment status			
Unemployed	1	1	1
Employed	0.46(0.34–0.62)*	0.79(0.61–1.04)	0.64(0.52–0.79)*
Schooling			
No	1	1	1
1–6 years	0.36(0.26–0.50)*	0.52(0.40–0.68)*	0.44(0.35–0.55)*
7–9 years	0.11(0.05–0.27)*	0.17(0.09–0.34)*	0.29(0.19–0.43)*
10 years or more	0.02(0.00–0.11)*	0.14(0.07–0.28)*	0.26(0.17–0.39)*
*P-trend*	<0.01	<0.01	<0.01
Smoking			
Currently non-smoker	1	1	1
Current smoker	1.24(0.85–1.82)	1.00(0.72–1.40)	1.08(0.84–1.39)
Drinking			
Currently non-drinker	1	1	1
Current drinker	1.30(0.89–1.90)	1.18(0.86–1.63)	1.06(0.82–1.36)
Medical insurance			
National Health Insurance	1	1	1
Medical Aid Program	5.02(2.37–10.63)*	2.18(0.89–5.35)	5.58(3.13–9.95)*

* P-value<0.05.

## Discussion

We investigated the prevalence of cognitive impairment, depression, and their comorbidity and factors associated with them among participants aged in their early 60s in a rural area of Korea. This may be the first study focusing on mental health problems and the comorbidity of these two conditions in subjects aged in their early 60s in Korea. The overall prevalence of cognitive impairment (MMSE-K ≤24) was 17.4%, that of depression (GDS-15 ≥8) was 26.0%, and that of comorbidity was 7.1%. Significant associations of gender, employment state, years of schooling, and GDS-15 score with cognitive impairment were observed. Marital status, employment state, years of schooling, type of medical insurance, and MMSE-K score were related to depression. The factors related to comorbidity were gender, marital status, employment status, years of schooling, and type of medical insurance.

Results from a study that employed only the MMSE-K to estimate the prevalence of cognitive impairment among people ≥65 years of age showed a prevalence (17.0%) [17] similar to our results (17.4%). Our results regarding the prevalence of depression were in the range of previous studies [3]. Variations in the prevalence of depression from previous studies conducted in Korea (9.1–33.0%) [3] may be due to differences in population characteristics such as education level.

Education is strongly related to the risk of developing dementia [18]. This effect is prominent in the Korean population compared with that in other countries, perhaps due to the generally lower education level among elderly Koreans and the consequent life-long lack of exposure to stimulating environments and accessibility to health services [17]. Lower education level can hinder the early development of brain function [19] and affect the absolute levels of cognitive function [20], and intellectual challenges may increase the brain reserve and delay development of dementia [18]. The higher OR for cognitive impairment in females in our results was consistent with previous results [21]. Gender-related differences in social exposure may have caused this difference in cognitive function [22]. Cognitive impairment increases the prospective risk of unemployment [23]. However, our result showing an association between employment and a low prevalence of cognitive impairment may be explained in another way. Given that the cross-sectional design of our study cannot show causal inference, we could also assume that loss of engagement in social activities caused by being unemployed could contribute to a decline in cognitive function [24], [25]. A study conducted in Korea showed that living with a spouse was related to a reduced likelihood of dementia [26], but in our results, although marital status was not associated with the presence of cognitive impairment, living with one companion was associated with a higher OR for the presence of cognitive impairment. This could also be explained by reverse causality; that is, perhaps those with cognitive impairment need the help of a caregiver, and living with a housemate may be the result rather than a cause of cognitive impairment.

A meta-analysis suggested that bereavement, sleep disturbances, disabilities, history of depression, and female gender were risk factors for depression among elderly individuals [27], and another study reported that less education was associated with depression in later life [28]. Ours results were consistent with those of other studies with regard to bereavement and education, but female gender was not related to depression in our study. Not only financial dependency but also financial dissatisfaction is associated with depression [29]–[31]. The type of medical insurance was a surrogate indicator of income in our study, as the Medical Aid Program is a medical support system for poor people. Beneficiaries of the Medical Aid Program showed a higher prevalence of depression. Depression has been found to be associated with unemployment and a loss of income in young adults [32], and our results showed a similar relationship between employment status and depression in participants in their early 60s.

We found an independent association between cognitive impairment and depression after adjusting for sociodemographic factors. Many studies have shown a consistent association between the two [17], [33], [34], and it has been suggested that late-life depression, cognitive impairment, and dementia could represent a possible clinical continuum [35]. It is possible that shared biological risk factors or interactions between coexisting factors might lead to increased risk of both cognitive impairment and depression [34]. The correlated factors for comorbidity of cognitive impairment and depression were similar to those for cognitive impairment and depression individually. Particularly, employment status and years of education were associated with all three conditions. A previous study showed that only female gender was a common risk factor for cognitive impairment, depression, and the comorbidity of the two conditions, but the sample size in that study was smaller [4] than that in our study.

This study has some limitations. We used a cross-sectional design, which is inappropriate for drawing causal inferences, so the results should be interpreted with caution. Second, generalization of the results should be made carefully, as the representativeness of the participants was limited, although most of the results were consistent with previous studies. Although the tools we used to measure cognitive impairment and depression have been validated in the Korean population, they are screening tools, and a measurement bias due to actual disease status may have occurred. Other mental health problems, for example anosognosia or anxiety which we did not measure might affect the score. Also, we measured only a limited number of variables and it is possible that an unmeasured trait was associated with cognitive impairment or depression. Finally, a nonresponse bias may have occurred. We did not examine the features of those who refused to participate and, thus, cannot estimate the extent of such bias. We suggest that any such effect may have been minimal, considering the high response rate.

Despite these limitations, this study has several strengths. We investigated the factors associated with the comorbidity of cognitive impairment and depression, which have been rarely studied despite their clinical importance. Additionally, the study population consisted of subjects in their early 60s whose mental health has been rarely investigated even though they are entering an important “presenile” stage.

## Conclusions

We investigated correlated factors for cognitive impairment, depression, and comorbidity of the two conditions, and we found that the associated factors were similar among the three conditions through an exploratory way. Among the correlated factors, employment status and years of schooling were particularly strongly associated with all three conditions. The shared correlated factors or interactions between coexisting factors might lead to increased risk of both cognitive impairment and depressive symptoms. Considering the greater decline and increased risk for adverse outcomes among those with comorbid depression and cognitive impairment, it could be suggested that patients with either of these mental conditions should be evaluated for other mental conditions.
